# Robust self-propulsion in sand using simply controlled vibrating cubes

**DOI:** 10.3389/frobt.2024.1298676

**Published:** 2024-08-30

**Authors:** Bangyuan Liu, Tianyu Wang, Deniz Kerimoglu, Velin Kojouharov, Frank L. Hammond, Daniel I. Goldman

**Affiliations:** ^1^ Institute for Robotics and Intelligent Machines, Georgia Institute of Technology, Atlanta, GA, United States; ^2^ George W. Woodruff School of Mechanical Engineering, Georgia Institute of Technology, Atlanta, GA, United States; ^3^ School of Physics, Georgia Institute of Technology, Atlanta, GA, United States; ^4^ The Wallace H. Coulter Department of Biomedical Engineering, Georgia Institute of Technology, Atlanta, GA, United States

**Keywords:** vibration, granular media, robot, locomotion, modular robot, robophysics, DEM simulation

## Abstract

Much of the Earth and many surfaces of extraterrestrial bodies are composed of non-cohesive particulate matter. Locomoting on such granular terrain is challenging for common robotic devices, either wheeled or legged. In this work, we discover a robust alternative locomotion mechanism on granular media-generating movement via self-vibration. To demonstrate the effectiveness of this locomotion mechanism, we develop a cube-shaped robot with an embedded vibratory motor and conduct systematic experiments on granular terrains of various particle properties and slopes. We investigate how locomotion changes as a function of vibration frequency/intensity on such granular terrains. Compared to hard surfaces, we find such a vibratory locomotion mechanism enables the robot to move faster, and more stably on granular surfaces, facilitated by the interaction between the body and surrounding grains. We develop a numerical simulation of a vibrating single cube on granular media, enabling us to justify our hypothesis that the cube achieves locomotion through the oscillations excited at a distance from the cube’s center of mass. The simplicity in structural design and controls of this robotic system indicates that vibratory locomotion can be a valuable alternative way to produce robust locomotion on granular terrains. We further demonstrate that such cube-shaped robots can be used as modular units for vibratory robots with capabilities of maneuverable forward and turning motions, showing potential practical scenarios for robotic systems.

## 1 Introduction

Many terrestrial and extraterrestrial landmasses are composed of soft flowable material, like sand and snow. Among them, granular media consists of discrete solid particles that dissipate energy upon interaction. Such terrain poses locomotor challenges for conventional wheeled and tracked devices. To surmount this challenge, diverse robotic systems have been developed to produce controllable movement in granular materials. These systems encompass legged configurations (Liang et al., 2012; Qian et al., 2015; Zhong et al., 2018; Lee et al., 2022), limbless structures (Maladen et al., 2011; Marvi et al., 2014), and rover designs (Knuth et al., 2012; Shrivastava et al., 2020). A common issue faced by such robotic systems attempting locomotion within granular media lies in the potential transition of the granular terrain from a solid to a flowing state, which occurs when the force per unit area exerted by the robot exceeds its yield stress. The interaction between the robot body and the terrain often engenders a coupled relationship encompassing the robot’s movement, the resistive force the robot experiences, and the dynamic changing of the terrain state before, during, and after the robot’s movement. Particularly in cases involving shear-based locomotion, the granular material can aggregate into formations that act as obstacles, hindering the robot’s progress (Shrivastava et al., 2020).

A vibration-driven robot leverages periodic oscillations or rotations of internal components to generate motion throughout the entire body (Becker et al., 2011; Reis et al., 2013; Calisti et al., 2017; Golitsyna, 2018). In contrast to conventional wheeled, legged, or elongated limbless robots, vibration-driven robots offer a simpler and more compact body design, without the need for body deformation and exposed actuators or joints. This intrinsic feature effectively prevents potential damage to the robot arising from particle infiltration or obstruction of the actuation mechanisms. Further, such design allows for a greater contact area between the robot’s body and the terrain. Therefore, the yield strain and stress for supporting body weight and producing propulsion can be reduced; consequently, the risk of terrain collapse is mitigated.

Previous research has designed and studied vibration-driven robots that can locomote on various terrains, such as hard surfaces (Rubenstein et al., 2012; Notomista et al., 2019; Li et al., 2021), fluid surfaces (Cocuzza et al., 2021; Tarr et al., 2024), and amphibious environments (Tang et al., 2018; Wang et al., 2022). These vibratory robots typically utilize a consistent actuation principle, wherein vibratory actuators induce multi-directional movement through alterations in force orientation, and the accumulated movement direction is dictated by anisotropic friction or resistive forces. These forces stem from specific directional components of periodic internal forces or inherent body features (Calisti et al., 2017; Golitsyna, 2018). However, the performance of such a vibration-driven mechanism on granular media remains unexplored.

**FIGURE 1:**
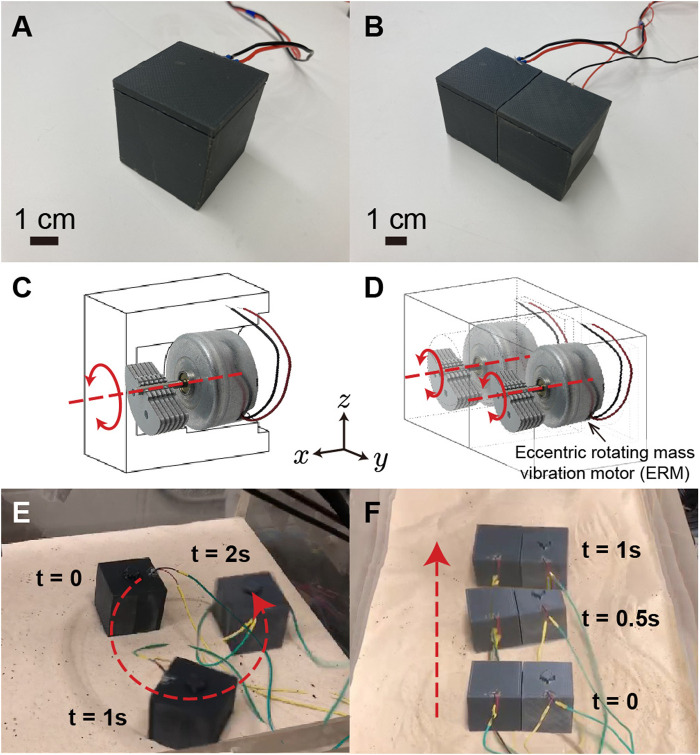
Vibratory single cube and bi-cube fabrication and motion. Panels **(A,C,E)** show the appearance, perspective drawing, and the turning maneuver of a single cube. By combining two single cubes together, the bi-cube is fabricated. Panels **(B,D,F)** show the appearance, perspective drawing, and the forward-moving maneuver of a bi-cube. The locomotion of single cube and bi-cube are recorded in Supplementary Video S1.

Due to its design simplicity, ease of control, and enlarged body-terrain contact area, a vibratory robot presents a promising avenue for achieving robust and effective locomotion on granular media. This work introduces the development of a novel vibrating robotic system (Figure 1) to investigate the locomotion capabilities of such vibratory mechanisms within different granular media and identify potential practical applications.

## 2 Materials and methods

### 2.1 Robot design and maneuver manipulation

#### 2.1.1 Single cube

For design simplicity, we used Solidworks to create a cubic box with an open side for the motor and a press-fit lid to close the cube (as shown in Figure 1). The 4-cm-side-length cube was then 3D printed using a LulzBot TAZ Workhorse 3D Printer and polylactic acid (PLA) as the printing material. To generate the vibration, we selected an eccentric rotating mass vibration motor (ERM), specifically the VJQ24 from Vybronics Inc. (motor weight 31 g, 2,550 rpm at 5 V DC). The rotary axis of the vibration motor lies horizontally in the *x* direction, parallel to the cube’s bottom surface. When power is applied, the uneven mass starts rotating, which leads to rotary oscillation about the *x*-axis. By switching the voltage from positive to negative, the vibration motor rotation direction converts from counter-clockwise to clockwise (view in the positive *x* direction), which allows the single cube and bi-cube to generate maneuvers. The position of the vibration motor is adjusted to the cube’s center, guaranteeing alignment between both the center of the cube’s mass and cube’s geometric center. Inside the cube, we implemented a structure to securely press fit the motor in place to ensure that it remains stationary while vibrating.

Influenced by the simultaneous lateral oscillatory inertia generated by the single cube system, the cube’s forward locomotion is accompanied by turning (shown in Figure 1). When the input voltage is positive, the counter-clockwise-rotating vibration motor induces a leftward turning. When the input voltage is negative, the clockwise-rotating vibration motor induces a rightward turning.

#### 2.1.2 Bi-cube and maneuver control

The bi-cube robot is comprised of two firmly bonded identical cubes by combining the cubes in the orientation in which the ERM’s rotary axes are parallel to each other. Such a bi-cube robot cannot only move forward but can also turn.

**FIGURE 2:**
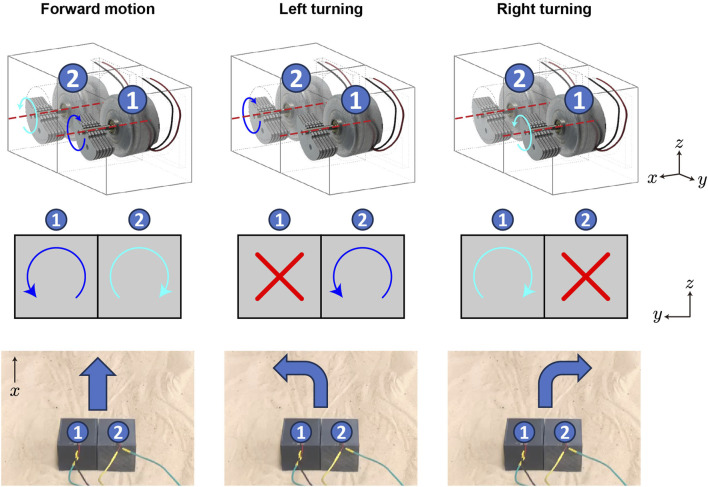
Bi-cube steering mechanism. The bi-cube can execute three maneuvers by controlling the rotational state of vibration motors #1 and #2 inside the left and right cubes: forward motion, left turning, and right turning, from the left column to the right column, with each row illustrating the motor state from the perspective view, back view, and the corresponding maneuver.

Based on the maneuver test shown in Figure 2, when the left cube (marked as #1 in Figure 2) vibration motor rotates counter-clockwise and the right cube (marked as #2) rotates clockwise, the lateral oscillation of each motor can be counterbalanced, thus the bi-cube robot performs a forward motion. When #1 cube turns off and #2 cube rotates counter-clockwise, the bi-cube robot performs a left turn. Similarly, when #2 cube turns off and #1 cube rotates clockwise, the bi-cube robot performs a right turn.

#### 2.1.3 Vibration frequency

**FIGURE 3:**
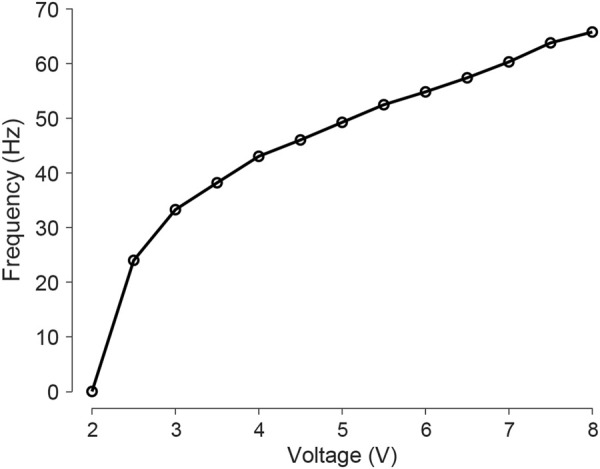
Vibration frequency of a cube hanging in air as a function of input voltage. The frequency increases from 0 Hz to 65 Hz when the voltage increases from 2 V to 8 V.

To correlate input voltage with the cube’s vibration frequency, we recorded acceleration data from a single cube hanging from a thread at various voltage inputs using an IMU sensor, sampled at 200 Hz. The vibration frequency data was derived from the acceleration data through Fast Fourier Transform (FFT) analysis in Figure 3. As the voltage increases, the vibration frequency increases.

### 2.2 Experiment environment

#### 2.2.1 Air-fluidized testbed

**FIGURE 4:**
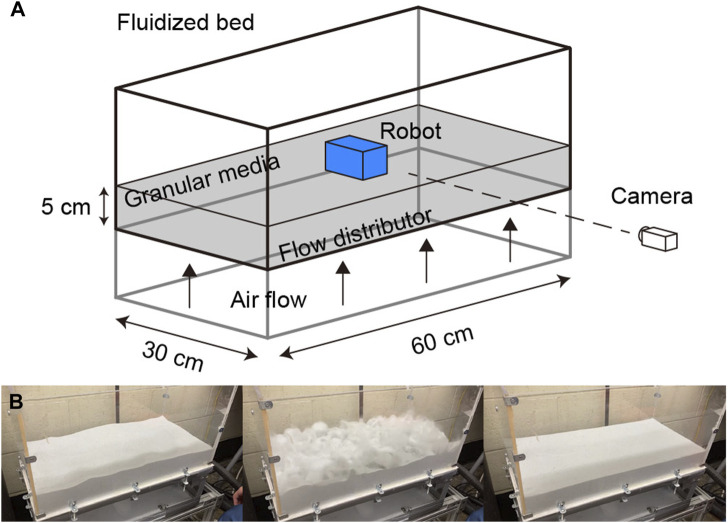
The automated terrain creation and locomotion testing system. **(A)** Diagram of the air-fluidized bed for robot locomotion testing. **(B)** The process of preparing the granular media before each experiment and creating a loosely packed state (left: before, mid: blowing air to the fluidized bed, right: loosely packed state achieved after cessation of air flow).

To ensure consistent initial conditions for our experiments on granular media, we implemented a terrain creation and locomotion testing system (as shown in Figure 4), following the approach described in previous work (Qian et al., 2013). The system uses an air-fluidized bed, a container (60 cm long, 30 cm wide) filled with granular material (5 cm deep) such that the surface can be flattened at the beginning of each experiment by blowing air through a porous rigid plastic layer. A vacuum (Vacmaster) is used to force air through the bottom of the bed. The flow distribution layer, which evenly distributes the air across the bed, is approximately 0.5 cm thick and has randomly distributed pores with a diameter of 50 μm. This allows for precise control over the bed’s surface and ensures that the initial conditions of each experiment are consistent; in these experiments all states are of loosely packed granular media (Qian et al., 2013).

#### 2.2.2 Granular materials

We expect that the locomotor capabilities of our robotic cube can vary depending on the type of granular material it is tested on. To investigate this, we tested robot speed and energy use on three different types of granular material: glass particles with an average diameter of approximately 200 μm (Figure 6A), sand with particle sizes ranging from 500 to 700 μm (Figure 6B, fine sand), and 1,000–1,200 μm (Figure 6C, coarse sand). The corresponding angles of repose for these materials are 18.6°, 28.8°, and 36.7°, which indicates different values of inter-grain friction. As a comparison, we also tested cube locomotion on a hard wooden board (Figure 6D).

### 2.3 Experimental protocol

For each of the four terrains we considered, we conducted a series of experiments with the bi-cube robot. We varied the input voltage from 0 to 8 V to test forward speed and cost of transport (CoT). Specifically, we limited the robot’s rotational movement using two parallel walls that were 10 cm away from each other (slightly larger than the robot’s width 8 cm). For each voltage input value, we carried out three repeated experiments. In each experiment, we placed the robot at one end of the tank ( > 5 cm away from the wall) and recorded the robot’s locomotion from when the robot started vibrating to when the robot reached the other end of the tank. For experiments where the robot was unable to reach the other end of the tank, we kept the robot running for at least 30 s. To capture the robot’s locomotion trajectory and orientation, we implemented a motion tracking system (OptiTrack) using 4 OptiTrack Flex 13 cameras. We attached infrared reflective markers to the robot body and tracked their 3D positions using the system at a 120 fps, thus the progress of the locomotion could be fully reconstructed and analyzed. We tracked the rigid body position and orientation in the world frame to calculate the forward speed. To calculate the cost of transport, we measured the power consumption of the system using an INA260 precision digital power sensor that monitors power at 100 Hz.

### 2.4 Discrete element method (DEM) simulation of a single cube

To enhance our understanding of cube movement on granular materials, we utilized Discrete Element Method (DEM) simulations, a methodology used in prior investigations into the physical interactions between robots and granular terrains Zhang et al. (2013); Pullinz et al. (2013). We used the open-source software Large-scale Atomic/Molecular Massively Parallel Simulator (LAMMPS) Thompson et al. (2022) to represent the motion of a vibrating single cube. To capture normal forces in granular media interactions during simulation, we used spherical particles in conjunction with the Hertzian contact model, where the normal force between two overlapping particles is proportional to the overlapping area. To model tangential forces between particles we used Mindlin No-slip solution. We incorporated rolling friction between particles, which is computed using a spring-dashpot-slider model. The parameters defining the granular particles in the simulations are detailed in Table 1, where we align the particle density and size distribution with the coarse sand particles. The simulation box of particles was created by randomly generating and pouring them into the bed. To construct the cube in simulation, we utilized 1 mm diameter spherical particles and connected them rigidly to form a hollow cube with the same dimensions as the actual cube Figure 11A). Inside the cube, a single particle is positioned at a distance d from the cube’s center of mass (CoM), modeling the eccentric mass of the vibration motor. We then applied internal sinusoidal forces to this particle in the vertical (z) and out-of-plane (y) axes, resembling the rotating interaction force F induced by the centrifugal force from mass rotation. The $F_{y}$ and $F_{z}$ are respectively as follows:

$$\begin{array}{cc} F_{z} & =m\omega^{2}rsin\left(\omega t\right)\\ F_{y} & =m\omega^{2}rcos\left(\omega t\right) \end{array}$$ (1)

**TABLE 1:** Parameters used for the DEM simulations.

Property	Value
Contact model	Hertzian
Particle diameter	0.9–1.4 mm
Particle density	1,500 kg/ ${\text{m}}^{3}$
Young’s modulus	$5\times 10^{6}$ Pa
Poisson’s ratio	0.3
Coefficient of restitution	0.2
Coefficient of friction (Normal)	0.6
Coefficient of friction (Rolling)	0.2
Timestep	$5\times 10^{-6}$ s

Here, m is the eccentric mass of the motor, ω is the angular frequency and r is the distance of the eccentric mass to the rotation axis. To calibrate the simulated cube’s motion against the experiments we used the forward locomotion dynamics of the experimental cube since we are interested in the horizontal displacement of the cube. We first used the horizontal (x-axis) oscillation frequency of the experimental cube as the excitation frequency in the simulation. Then, using vibration motor kinematics and Eq. 1, we obtained the force amplitude and applied it in the simulation to closely resemble the experimental data shown in Figure 9. The cube simulation was conducted for a duration of 1 s.

## 3 Result and discussion

### 3.1 Locomotor performance

#### 3.1.1 Forward velocity

We measured the average speed under various applied voltages to quantify the bi-cube robot’s locomotion performance.

**FIGURE 5:**
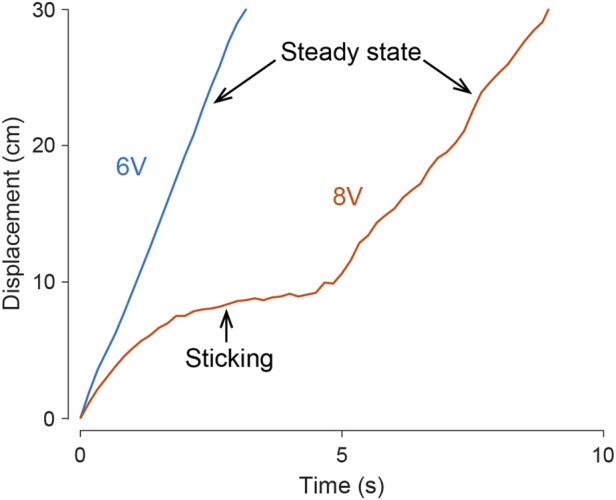
Steady-state motion and a sticking case in bi-cube locomotion on find sand. In various test conditions and trials, the bi-cube robot demonstrates smooth movement, as depicted by the blue curve, illustrating a motion tracking trial under a 6 V input. In challenging scenarios, such as navigating loosely compacted terrain pits (illustrated by the red curve under an 8 V input), the robot may encounter temporary impediments before successfully escaping and achieving a stable, steady-state motion. The averaged velocity in Figure 6 is computed by the steady-state motion.

**FIGURE 6:**
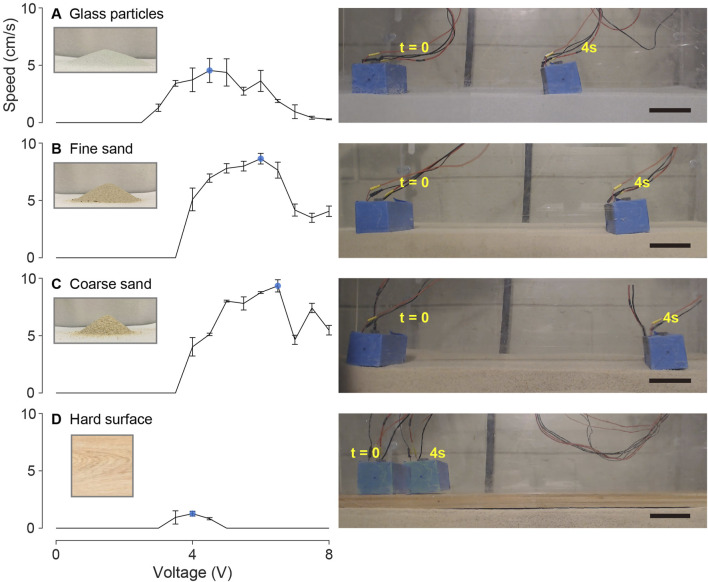
Bi-cube speed test on **(A)** glass particles, **(B)** fine granular media (fine sand), **(C)** coarse granular media (coarse sand), and **(D)** hard wooden surface. Each figure in the left column shows the averaged velocity of three experiment trials as a function of input voltage from 0 to 8 V, with the error bar showing the standard derivation. The local maximum velocity on each terrain is marked by the blue dot. The particle pile image shows the angle of repose of glass particles (18.6°), fine sand (28.8°), and coarse sand (36.7°). The right column shows an example locomotion of the robot operated with the optimal input voltage in each terrain, in which the frames are recorded at t = 0 and t = 4s. The black scale bars indicate 5 cm. The performance is shown as videos in Supplementary Video S2.

Under specific voltage input conditions, as illustrated in Figure 5 with the 8 V input on fine sand, the bi-cube robot may unpredictably become stuck in a collapsed pit (bubble remnants from rapid solidification), particularly during the initial phase of each test trial. Once freed from the pit, the robot exhibits steady-state motion. Utilizing tracked data, we computed the average forward speed (measured in cm/s) achieved by the bi-cube robot during each experiment’s steady-state motion. The values were averaged over three separate trials, as depicted in Figure 6, where error bars represent the standard deviation.

In glass particles, the bi-cube robot cannot generate effective locomotion when the input voltage is less than 3 V. With increasing input voltage, the forward locomotion speed increases, reaching the local maximum of 4.55±1.05 cm/s at 4.5 V. As the input voltage steadily increases, reaching the maximum voltage of the ERM at 8 V, performance gradually declines. Comparable trends in locomotion performance are observed in fine granular media (fine sand) and coarse granular media (coarse sand) as well. However, the minimum input voltage required for effective motion increases to 4.5 V in these cases. Moreover, local maxima in forward speed emerge at higher input voltages. Specifically, in fine granular media, the local maximum is observed at 8.64±0.46 cm/s with an input voltage of 6 V. In the case of coarse granular media, the local maximum reaches 9.34±0.54 cm/s with an input voltage of 6.5 V. For comparative analysis, we conducted locomotion performance tests on a hard wooden surface. The results illustrate that only a narrow input voltage range (from 3.5 V to 4.5 V) yields forward movement for the bi-cube robot on this terrain. Further, the maximum speed achieved is only 1.27±0.21 cm/s at 4 V, slower than the velocities achieved in granular media.

Through these experimental findings, we demonstrate that the bi-cube robot is capable of achieving effective locomotion across various granular media types (at rates greater than 1 body length per second). Furthermore, we observed that the optimal operating voltage increases with the grain size and coefficient of friction. This optimal voltage may be influenced by factors such as robot dimensions or hardware limitations. These limitations encompass actuator output power and vibration intensity, as well as the unsteady desynchronization between the two vibration motors in the high-voltage input region.

#### 3.1.2 Locomotion efficiency

**FIGURE 7:**
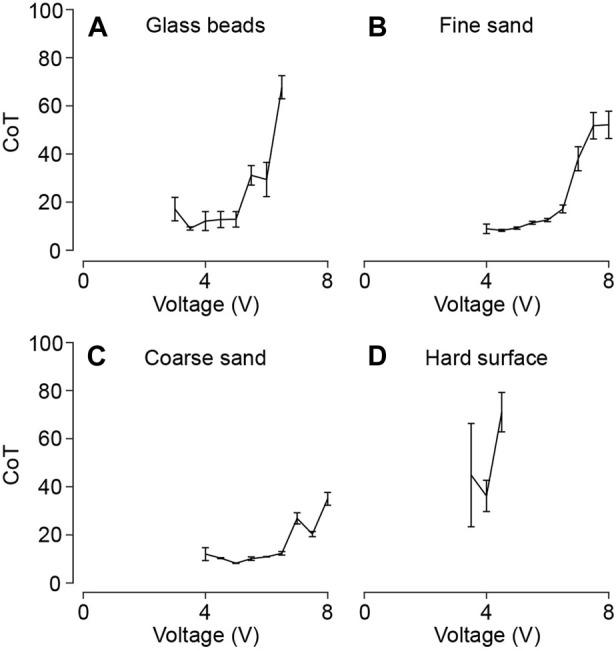
Bi-cube locomotion cost of transport (CoT) test on **(A)** glass particles, **(B)** fine granular media (fine sand), **(C)** coarse granular media (coarse sand), and **(D)** a hard wooden surface. Each figure shows the averaged cost of transport (CoT) of three trials as a function of input voltage with the error bars showing the standard derivation. Data points with extremely large CoT values (≫100) are omitted since the robot is unable to achieve effective locomotion, which result in a cost of transport exceeding 100.

In addition to evaluating the forward locomotion speed, we conduct real-time power consumption measurements for each experiment. Subsequently, we calculate the cost of transport (CoT) following $\text{CoT}=\frac{P}{mgv}$ , where P and v represent the average power consumption and speed achieved during the locomotion process, respectively, and m is the mass of the bi-cube robot (147 g). Figure 7 shows CoT values of the bi-cube robot traversing four different types of terrain that we tested.

Experimental results reveal that the local minima of CoT vary corresponding to grain sizes and coefficient of friction. Notably, these local minimum cost of transport values remain below 10 across all granular terrains: 9.06±0.66 at 3.5 V in glass particles, 8.23±0.42 at 4.5 V in fine granular media, and 8.31±0.09 at 5 V in coarse granular media. However, we notice that the local minima of the cost of transport do not coincide with the local maxima of speed. Note that we have excluded data points corresponding to input voltages where the robot is unable to achieve effective locomotion, resulting in a cost of transport exceeding 10. Our findings demonstrate that the bi-cube robot exhibits more efficient locomotion within granular media in comparison to hard surfaces. This highlights its potential for broader applications within granular environments.

In comparison, we surveyed available literature values of the cost of transport (CoT, including all work input to the system) or the mechanical cost of transport (mCoT, ignoring internal wasted work) for some other living creatures and robots locomoting on dry granular terrain, such as sandfish (CoT: 100), shovel-nosed snake (CoT: 50) (Hosoi and Goldman, 2015), limbless robot (mCoT: 5-15) (Wang et al., 2023), bipedal rover (mCoT: 20-200), quadrupedal rover (mCoT: 10-100) (Bagheri et al., 2023), and Bipedal walking (mCoT: 0.67) (Hosoi and Goldman, 2015). The vibrating cube’s CoT falls within a reasonable range compared to other systems at the same scale. Furthermore, as we measure the overall cost of transport rather than the mechanical cost of transport, the vibrating cube’s mCoT value is anticipated to be smaller.

#### 3.1.3 Voltage difference impact on forward maneuver velocity

**FIGURE 8:**
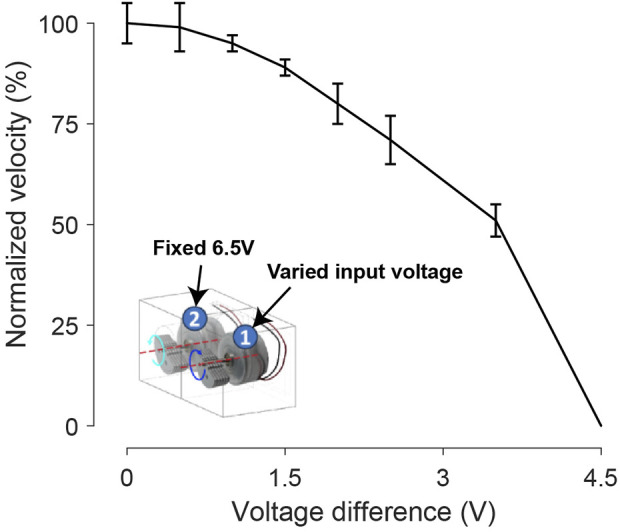
The influence of a voltage difference to the two motors of the bi-cube on the forward velocity. During each test, we fixed motor 2 input at 6.5 V and varied motor 1 input from 6.5 V to 2 V. The corresponding voltage difference increases from 0 V to 4.5 V. Each velocity is tested 3 times repeatedly, with bars representing standard deviation.

The forward maneuver necessitates maintaining an equal input voltage magnitude for both Cube #1 and Cube #2. Any divergence in voltage between the two sides adversely affects maneuver performance and, with increasing divergence, eventually leads to turning. In Figure 8, we explore the impact of input voltage divergence on the forward motion velocity component. Specifically, we applied a fixed voltage input (6.5 V) to one side’s vibration motor while varying the voltage on the other side. As the voltage difference increases, the forward motion velocity component consistently decreases.

### 3.2 Motion recording via high speed imaging

To gain insight into the mechanisms governing the bi-cube robot’s translation through vibration and subsequently develop either a kinematic or dynamic model to elucidate its motion, we utilized a high-speed 240 fps camera to capture the rigid body orientations of the robot. Subsequently, we extracted specific frames to calculate the instantaneous displacements in the forward (x) direction.

As discussed in Section 3.1.1, the inherent unsteady desynchronization issues identified in the bi-cube system can randomly disrupt the forward direction trajectory. Consequently, we opt to constrain the lateral motion of the single cube, focusing solely on tracking the forward direction component.

**FIGURE 9:**
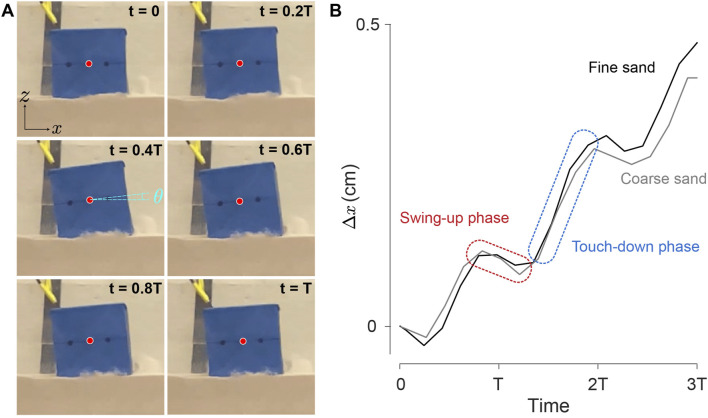
Single cube motion tracking. **(A)** A sequence of the robot posture in the abstracted 2D workspace over one complete period, the red dot represents the center of geometry. The corresponding video is provided in Supplementary Video S3. **(B)** Tracked trajectories of x displacement of the center of geometry on coarse and fine sand.

In Figure 9A, we depict a sequence of the robot’s postures during a motion period in the quasi-2D setup under a 5 V input on fine sand. Figure 9B illustrates the x displacements of the center of geometry for both fine sand and coarse sand at 5 V. The x trajectory displays periodic backward and forward motion behavior that correlates to two distinctive phases: the swing-up phase and the touch-down phase, which we illustrate in the following locomotion mechanism hypothesis Section 3.3.

Our observations revealed that the single cube exhibited a higher velocity than the bi-cube system. Specifically, in the context of the coarse sand forward speed component, the single cube (Figure 11E) demonstrated a velocity ranging from 8–14 cm/s as the voltage increased from 4 V to 8 V (where the excitation frequency simultaneously varies from 35 to 55 Hz). In contrast, the bi-cube system (Figure 6C) exhibited a lower speed, ranging from 4–9 cm/s. This discrepancy can be attributed to the lateral forces induced by the single cube, resulting in lateral shaking and, consequently, additional forward propulsion as its back corner anchors in the sand, complementing the turning effect. Conversely, the bi-cube system counterbalances such lateral forces, emphasizing its capability for maneuvering.

Moreover, we observed enhanced stability in the single cube, particularly in the high-voltage region (6.5–8 V). This increase in stability can be attributed to the avoidance of unsteady desynchronization issues inherent in the bi-cube system, as discussed in Section 3.1.1.

### 3.3 Locomotion mechanism hypothesis

**FIGURE 10:**
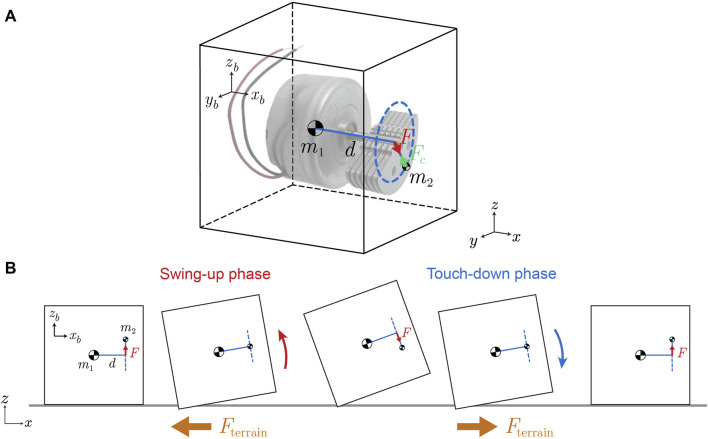
A simplified model for understanding single cube locomotion. **(A)** The 3D cube model. The vibration motor pendulum mass $m_{2}$ rotates in a circular trajectory around the shaft. Its centripetal force $F_{c}$ (marked by green arrow) induces an interaction force F (marked by red arrow) on the motor shaft, which points from the shaft to the $m_{2}$ center. The F rotates in a yz plane in the cube frame, which is perpendicular to the shaft, with a shaft distance d away from the main body mass $m_{1}$ . **(B)** The simplified 2D cube model. The bi-cube system cancels out the y axis horizontal component of oscillating force F .

The x trajectories in the experimental tracking data (Figure 9B) display periodic behavior with a distinctive pattern: over a single motion cycle, the robot undergoes a backward and forward motion which correlates to two distinct phases—the swing-up phase and the touch-down phase (Figure 10B). This periodic motion is induced by the oscillation of the vibration motor’s pendulum, located at the front part of the cube.

In the single cube 3D model (Figure 10A), the pendulum mass $m_{2}$ rotates in a circular trajectory around the shaft. In the cube frame $x_{b}y_{b}z_{b}$ , the centripetal force $F_{c}$ acting on it (denoted by the green arrow, pointing from the center of pendulum mass $m_{2}$ towards the motor shaft) generates a reaction force F (indicated by the red arrow) exerted on the motor shaft. This force F (which can be thought of as a centrifugal force acting on the motor shaft) points from the shaft towards the center of pendulum mass $m_{2}$ . In the cube frame, F rotates within the $y_{b}z_{b}$ plane at a specific frequency, perpendicular to the shaft, and acts at a distance d from the main body mass $m_{1}$ (which is approximately at the geometric center as designed and measured). Thus the cube experiences fluctuating forces and torques throughout a cycle.

As force F rotates within the single cube 3D model, it triggers oscillations along the $y_{b}$ and $z_{b}$ axes with a quarter-phase lag in the cube frame $x_{b}y_{b}z_{b}$ . In a bi-cube system, the opposing motor rotations on two sides counterbalance the oscillation along the $y_{b}$ axis, resulting in only the force component $F_{z}$ along the $z_{b}$ axis acting on the motor shaft. The 3D model can thus be simplified to a 2D cube model (Figure 10B). This vertical force component $F_{z}$ induces a periodic *z*-direction vertical motion and *y*-axis body rotation at the mass/geometric center. Additionally, as the cube rotates, the force component $F_{z}$ in the cube frame $x_{b}y_{b}z_{b}$ generates an *x*-axis force component in the world frame xyz , which induces slight back-and-forth oscillation in the x direction.

During the swing-up phase in each oscillation cycle, the cube elevates its front part and undergoes a counter-clockwise rotation (as shown in Figure 10B, left half part body diagram). The cube tends to push the sand beneath it in the positive *x*-direction and generate the negative *x*-direction force $F_{terrain.}$ When combined with the *x*-axis inertial force component of $F_{z}$ in the world frame xyz , this interaction generates a resistive force in the negative *x*-direction, pushing the cube backward. This results in a backward movement, which is indicated by the red dashed line in the *x*-direction trajectory in Figure 9B.

In contrast, during the touch-down phase, the front part of the cube drops down and rotates clockwise (illustrated in Figure 10B right half part body diagram). The cube tends to push the sand around its back bottom corner to the negative *x*-direction which leads to a positive *x*-direction force $F_{terrain.}$ When combined with the *x*-axis inertial force component of $F_{z}$ in the world frame xyz , this interaction generates a propulsion force in the positive *x*-direction, pushing the cube forward. This results in a forward movement, as indicated by the blue dashed line in the *x*-direction trajectory in Figure 9B.

During the swing-up phase, the cube is lifted by the upward direction force F . At the beginning of this phase, the cube’s back portion experiences increased normal forces. These forces presumably lead to increased resistive force due to penetration of the tip into the medium; such interactions help resist cube movement in the negative x direction. In comparison, at the beginning of the touch-down phase, the cube drops down from the air and inertially swings forward. When the cube hits the sand, the belly drag induced by the granular surface resistive forces rapidly brings the cube to rest. Because of the imbalance in the resistive forces during forward and backward oscillations, the forward swing results in a greater distance covered during the forward movement phase compared to the backward movement phase. Consequently, a net forward displacement is achieved per cycle.

### 3.4 Simulation analysis

**FIGURE 11:**
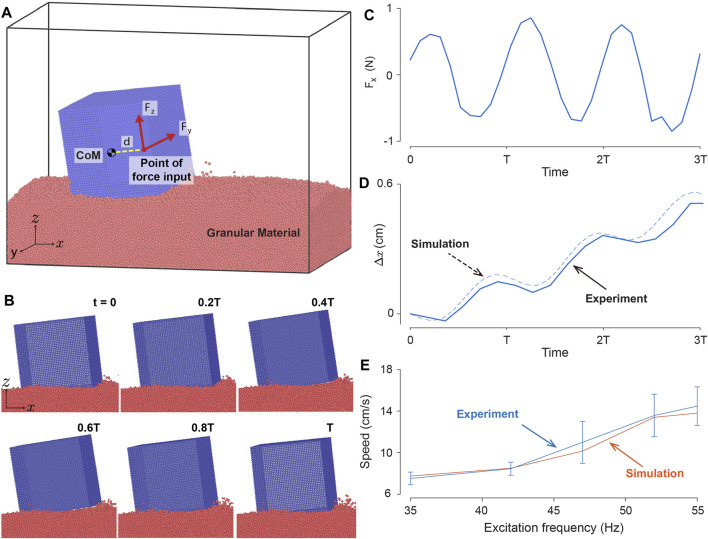
Single cube system DEM simulation. **(A)** DEM model of a single vibrating cube. External forces $F_{z}$ and $F_{y}$ are applied at a distance from the cube’s center of mass. **(B)** A sequence of the DEM simulation of the robot captured for one complete period. The excitation amplitude and frequency are 3.7 N and 42 Hz, respectively. The performance is recorded in Supplementary Video S4. **(C)** Forward net force induced by input force to the cube in the *x*-axis. **(D)** Example periodic trajectories of x displacement of the center of mass of the simulated and experimental (coarse sand, 5 V) single cube. **(E)** Single cube system DEM simulation across various excitation inputs. The experimental velocity is determined through three trials using a single cube on coarse sand, and the results are presented with a standard deviation bar.

We used DEM simulation to test the picture above and to determine if we could quantitatively reproduce the behavior of the single cube on coarse sand (see Figure 11A). By applying a rotating interaction force F , composed of $F_{y}$ and $F_{z}$ , on a particle offset by a distance d from the cube’s center of mass, the simulations demonstrate that the oscillatory dynamics of the experimental single cube can be recapitulated as illustrated in Figure 11B. To better illustrate the dynamics of the simulated cube, we first plot the net force acting upon the cube along the axis of the cube’s movement (*x*-axis) as depicted in Figure 11C. Here the external forces applied to the *y* and *z*-axes along with granular reaction forces indicate that the single-cube system is experiencing a net force along the *x*-direction. We then plot the horizontal displacement of the simulated cube’s center of mass, to draw a comparison with the experimental cube as depicted in Figure 11D. By approximating the forces from the vibration motor, the simulated cube closely replicated the oscillatory dynamics of the experimental cube.

To further test our model’s capabilities we compared the forward locomotion speed of the experimental cube with that of simulation across different excitation amplitude and frequencies on coarse sand as illustrated in Figure 11E. For comparison purposes, we used excitation frequency for both simulation and experiment where the voltage applied to the experimental cube is converted to excitation frequency as explained in Section 2.1.3. The simulations and experiments exhibit monotonically increasing speeds as the excitation frequency increases, suggesting that increased energy input to the cube yields faster locomotion. This result for a single cube is in contrast with the bi-cube system speed performance, depicted in Figure 6. We note that the performance mismatch between single and bi-cube systems might be partly due to the phase difference and slight difference in mechanics between the vibration motors in the bi-cube system. We leave investigating this disagreement in a future study. Nevertheless, the simulation results not only yielded displacement dynamics similar to those of the experimental cube but also exhibited similar speeds across various excitation profiles.

### 3.5 Payload test

**FIGURE 12:**
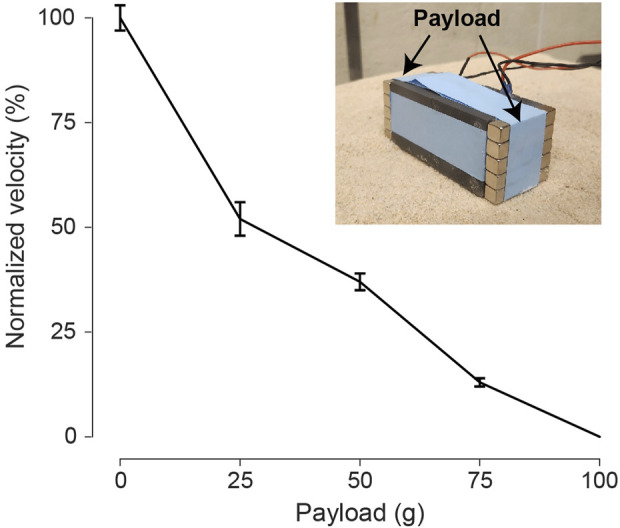
Payload test for the bicube during forward locomotion. The payload is set on two sides of the bi-cube evenly, ranging from 25, 50, 75, to 100 g. Compared to the 0-payload condition, normalized velocity is calculated with a standard deviation based on 3 repeated trials. The experiment is conducted on coarse sand at 6.5 V.

As payload capability is crucial for a robotic locomotion system, we assessed the bi-cube robot’s capacity by introducing additional weights to its body, as illustrated in Figure 12. To maintain the center of mass position, we evenly attached magnetic blocks to both sides of the robot. The original mass of the bi-cube is 146 g. As the payload increased, the forward velocity steadily decreased, eventually ceasing forward motion when the payload reached 100 g. The 146 g bi-cube demonstrated the ability to carry up to 75 g (approximately 50% of its original weight) payload while still moving, albeit at a slower speed. The payload capacity could potentially be enhanced with a more robust vibration motor.

### 3.6 Surface friction

**FIGURE 13:**
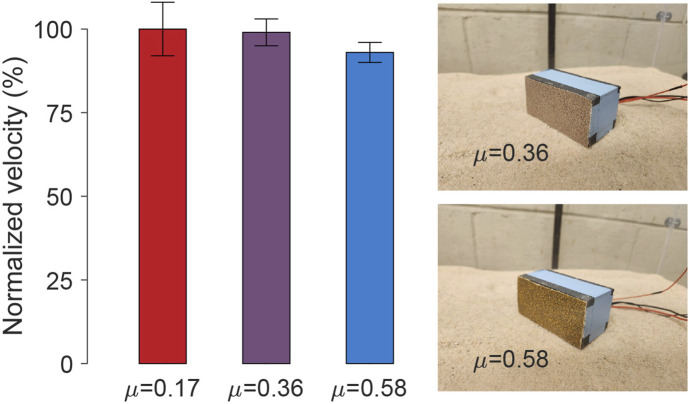
Robot-substrate contact surface friction test. The influence of cube surface friction on forward velocity is tested by attaching different sandpaper sheets to the bottom. The friction coefficients between the bi-cube surface and the wooden board are measured, which are 0.17, 0.36, and 0.58, respectively. The normalized velocity is calculated with a standard deviation based on 3 repeated trials. The experiment is conducted on coarse sand at 6.5 V.

According to the locomotion hypothesis in Section 3.2, the forward propulsion of the bi-cube motion primarily arises from the interaction with granular terrain and the particle jamming at the bi-cube’s bottom rear part and the inertial swing forward; the cube’s stoppage during each cycle should be dominated by the energy dissipated during swing down (due to multiple inelastic and frictional grain collisions). We thus expected that for a range of cube belly surface friction values, the locomotor performance should be insensitive to surface-grain friction. To test this, we conducted experiments measuring forward motion velocity with various surface friction coefficients. Different sandpapers were attached to the robot’s bottom, as illustrated in Figure 13. Since it was challenging to measure the friction between the cube and the environment, we characterized the belly resistance by measuring the friction coefficients between the bi-cube’s surface and the wooden board. The results (Figure 13) indicate that for the coarse sand, varying belly surface friction does not significantly impact velocity performance. We expect that for sufficiently low friction surfaces, performance could improve although this could be masked by the large dissipation due to the collision.

### 3.7 Slope climbing

Slope climbing remains challenging for many robots designed to navigate granular environments (Marvi et al., 2014; Shrivastava et al., 2020), as steep granular slopes tend to be sensitive to stress and shear forces disturbances (Tegzes et al., 2003; Gravish and Goldman, 2014), which can cause avalanche. The intricate nature of these terrains becomes particularly evident during climbing maneuvers, as even minor disturbances introduced by robot motion can trigger avalanches and instigate the transition of the terrain from a solid to a fluid-like state, resulting in failure.

**FIGURE 14:**
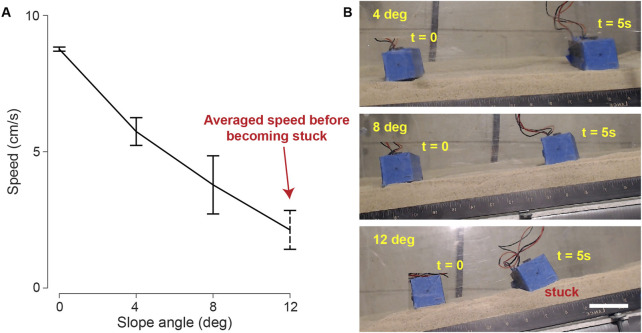
Bi-cube slope climbing. **(A)** The averaged velocity of three trials versus slope angle with standard derivation bars. The cube is tested on coarse granular media (coarse sand) at 6 V. At 12-degree slope, we recorded the average speed before the bi-cube got stuck. **(B)** The bi-cube slope climbing motion (4, 8, 12°), recorded at frames of t=0 s and t= 5 s. The scale bar is 5 cm. The performance is recorded in Supplementary Video S5.

To investigate the slope-climbing capabilities of the bi-cube robot, we conducted tests on the testbed with coarse sand, with one side of the testbed elevated, thereby forming granular slopes with 4, 8, and 12 degrees of inclined angle (Figure 14B). Figure 14A shows the average speed and standard deviation of the cube’s motion, powered by 6 V input voltage, averaged over three trials. With increasing slope angles, there was a continuous decrement in the cube’s velocity. At a 12-degree slope angle, we observed that the granular slope would sometimes collapse during robot locomotion. Consequently, the robot would become stuck shortly after climbing a few body lengths.

### 3.8 Escaping from entrapment

Given that the testbed was fluidized before each experiment, the loosely compacted granular terrain could unpredictably collapse during robot locomotion (due to the inherent bubbling in fluidized beds of this particle size Anderson et al., 1995). This led to the formation of small pits in the terrain which could cause the robot to get stuck. Thus we conducted experiments to test the bi-cube robot’s ability to escape from pits. Through experiments, we verified that with appropriate input voltages (e.g., 5 V in fine granular media), the bi-cube robot could extricate itself from a pit: The robot first engaged in a process of crawling, gradually moving the sand pile from its front to rear; and eventually, this enabled the robot to successfully escape entrapment. We provide a demonstration in Supplementary Video S6, in which the robot first became stuck in a pit in fine granular media (fine sand) when actuated by 3 V, and escaped with a 5 V input.

### 3.9 Maneuverability test

**FIGURE 15:**
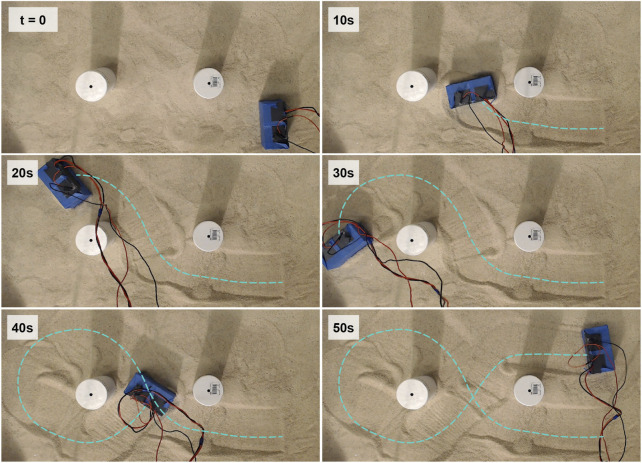
Bi-cube maneuver demonstration. The cube moves in an “ α ”-shaped trajectory (marked by the cyan dash line) around cylinder obstacles. The cube maneuver is manually controlled (switched between maneuvers as shown in Figure 2). Each frame records the cube position at t=0,10,20,30,40,50 s.

The bi-cube robot exhibits the capacity to execute forward, left, and right turning maneuvers, enabling effective navigation across 2D granular terrains. We illustrate this capability through a maneuver demonstration conducted on a coarse granular media surface. Notably, two cylindrical obstacles have been rigidly placed within the terrain, as depicted in Figure 15. In this demonstration, the robot’s maneuvering actions are manually switched among forward moving, left turning and right turning as shown in Figure 2. The cube follows an “ α ”-shaped trajectory, avoiding any potential collisions with the cylinder obstacles. We provide Supplementary Video S7, which describes the bi-cube robot’s agility and maneuverability while traversing complex granular terrains.

## 4 Conclusion

In this paper, we systematically tested the capability of vibratory locomotion on granular media in experiments and simulations. The vibration cube exhibits the capability to navigate across granular terrains of various particle sizes as well as solid ground. Notably, this vibratory locomotion method performs better on granular media surfaces than on hard ground, excelling in both velocity and efficiency. We posit that the flowable nature of the granular terrain plays a pivotal role in stabilizing motion and attenuating extraneous vibration energy, thereby amplifying locomotive efficacy. The inherent vibratory locomotion mechanism showcases an inherent affinity for granular terrains, suggesting a harmonious alignment between the mechanism and such environments. We employed DEM simulations to replicate the locomotion patterns of a vibrating single cube on granular particles. The simulation outcomes corroborated the experimental observations, demonstrating that the cube attains forward motion through interplay of centrifugal forces, torques and rotations generated by the vibration motor and its interaction with the granular media.

A single cube only demonstrates the capacity to execute left and right turns. However, through the fusion of two individual cubes, a bi-cube configuration is achieved, facilitating forward, left, and right turning. This amalgamation imparts a notable enhancement to maneuverability. Moreover, the inherent simplicity of the vibration cube underscores its potential to exhibit swarming capabilities on granular terrain.

Future work includes revealing the granular vibratory locomotion mechanism via both comprehensive theoretical modeling and experimental validation. Further, we intend to conduct investigations into the influence of particle size and density on locomotion efficiency based on simulation. Additionally, we will upgrade the vibration cube into a swarm robotic system, integrating self-feedback loops and inter-unit communication, for potential future application in exploration.

## Data Availability

The raw data supporting the conclusions of this article will be made available by the authors, without undue reservation.
